# First records of Cotylea (Polycladida, Platyhelminthes) for the Atlantic coast of the Iberian Peninsula

**DOI:** 10.3897/zookeys.404.7122

**Published:** 2014-04-22

**Authors:** Carolina Noreña, Daniel Marquina, Jacinto Perez, Bruno Almon

**Affiliations:** 1 Dept. Biodiversidad y Biología Evolutiva. Museo Nacional de Ciencias Naturales (CSIC). Calle Jose Gutierrez Abascal 2, 28006 Madrid. Spain; 2 Grupo de Estudos do Medio Mariño (GEMM), Puerto deportivo s/n 15960 Ribeira, A Coruña, Spain; 3 Instituto Español de Oceanografía, Canary Islands, Centro Oceanográfico de Canarias, Vía Espaldón, parcela 8, 38180 Santa Cruz de Tenerife, Spain

**Keywords:** Euryleptidae, Prosthiostomidae, Pseudocerotidae, new species, Spain

## Abstract

A study of polyclad fauna of the Atlantic coast of the Iberian Peninsula was carried out from 2010 to 2013. The paper reports nine new records belonging to three Cotylean families: the family Euryleptidae Lang, 1884, Pseudocerotidae Lang, 1884 and the family Prosthiostomidae Lang, 1884, and describes one new species, *Euryleptodes galikias* **sp. n.**

## Introduction

As a result of studies by Bock (1913), Faubel (1983/1984), Faubel and Warwick (2005), Prudhoe (1985) and Hyman (1940), the North European and North American polyclad fauna of the Atlantic coast is relatively well known. Therefore, sufficient information is available to establish, or recognize, zoogeographical distribution patterns for polyclads in the Atlantic region. The five regions proposed by Prudhoe (1985) are: 1. the Eastern North Atlantic cold-temperate or eastern boreal province, which extends from northern Norway to the English Channel; 2. the Lusitanean province, or North Atlantic warm-temperate region, which comprises the area from the southern part of the English Channel to the Black Sea coasts, including the Mediterranean Sea and the Cape Verde Islands; 3. the Western boreal province, comprising the northern west coast of the Atlantic Ocean, the coasts of Canada and the United States from Bat Baffin (Canada, Arctic Circle) to Cape Cod (New England, United States); 4. the Carolinian province, extending from south of Cape Cod to North Carolina; 5. the West Indian province encompasses the Gulf of Mexico to southern Brazil, including the Antilles and Bermuda, characterized by warm tropical waters.

Some Mediterranean species, such as *Prostheceraeus rubropunctatus* Lang, 1884; *Prostheceraeus vittatus* (Montagu, 1815) Lang, 1884; *Prostheceraeus roseus* Lang, 1884; *Anonymus virilis* Lang, 1884; or *Oligocladus sanguinolentus* (Quatrefages, 1845), are also found in the Cape Verde archipelago or along the European coast, but there are no records for these species for the American Atlantic coast. Except for species that have a nearly worldwide distribution, such as *Thysanozoon brocchii* (Risso, 1818) Grube, 1840) or *Stylostomum ellipse* (Dalyell, 1853) Lang, 1884, there are no species shared between the eastern and western coast of the Atlantic Ocean.

Temperature seems to form a natural barrier along the longitudinal axis of the Atlantic Ocean. The distribution of polyclads follows a north-south pattern for both the east and west coasts. Therefore, there are species common to both the Mediterranean and Scandinavian coast, but not to the American and European coasts.

In this paper, we present nine new records belonging to three Cotylean families: the family Euryleptidae Lang, 1884, Pseudocerotidae Lang, 1884 and the family Prosthiostomidae Lang, 1884, and we describe one new species, *Euryleptodes galikias* sp. n.

## Material and methods

Polyclads were found off the coast of northwestern Spain (Galicia) by scuba diving, while cataloguing the marine fauna of Ria de Arosa (NW Spain) under the auspicies of the Grupo de Estudo do Medio Mariño ("Study Group of the Marine Environment" GEMM), which has been operating for the past 10 years. These surveys cover a bathymetric range from the intertidal zone to a maximum accessible depth for scuba diving of approximately 40 meters. Most species were found in habitats typical for polyclads e.g., empty mollusc shells, over Bryozoa, algae, etc.

Specimens were first photographed in the field, then collected by hand using a brush or net and stored in containers according to specimen size. Once in the laboratory, a small piece of tissue was carefully removed for DNA analysis, and the rest of the animal was fixed with Bouin’s fluid for a complete histological study of its anatomy. The specimens were dehydrated in alcohol, cleared in xylene and subsequently photographed to record details about the eyes, pigmentation, body shape and tentacles, as well as the location of the pharynx and reproductive organs. Afterwards, they were embedded in paraplast, sagittally sectioned at 7 µm and stained with Azan trichrome. Reconstructions of internal morphologies were derived from serial sagittal sections. Measurements were determined from fixed material.

The material was deposited in the Invertebrate Collection of the Museo Nacional de Ciencias Naturales (MNCN; Spain).

Abbreviations used in the figures

b brain

ce cerebral eyes

cg cement glands

cp cement pouch

ed ejaculatory duct

esv external seminal vesicle

fa female atrium

fe frontal eyes

fp female pore

i intestine

ib intestinal branches

ma male atrium

mp male pore

o ovaries

op oral pore

p papillae

ph pharynx

pv prostatic vesicle

rh rhabdites

s stylet

spb spermiducal bulbs

su ucker

sv seminal vesicle

t tentacle

te tentacular eyes

ts testes

uv uterine vesicles

v vagina

vd vas deferens

## Systematics

### Suborder COTYLEA Lang, 1884

#### Family EURYLEPTIDAE Lang, 1884

##### Genus *Cycloporus* Lang, 1884

###### Cycloporus papillosus

(Sars, 1878) Lang, 1884

http://species-id.net/wiki/Cycloporus_papillosus

Figure 1

####### Material examined.

Four individuals captured during summer, autumn and winter between 2010 and 2012 (11/10/2010; 03/08/2011; 27/01/2011; 09/01/2012). Voucher: one specimen sectioned sagittally, stained with azan and deposited in the Invertebrate Collections of the MNCN; Cat. Nr: MNCN 4.01/573 to 4.01/599 (27 slides). Further material: one specimen cat. Nr. MNCN 4.01/600 to 4.01/625 (26 slides).

####### Description.

**Figure 1.**
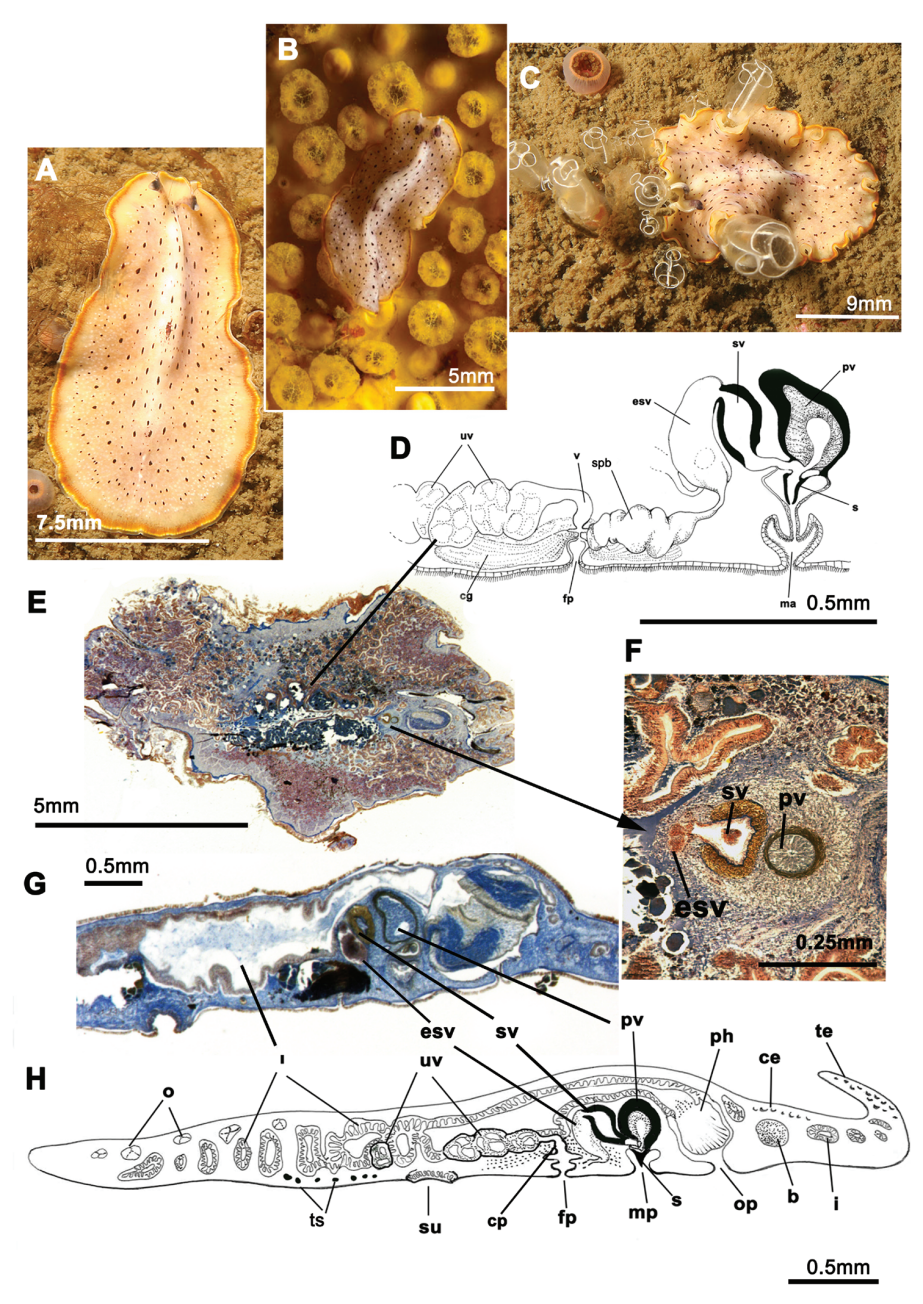
*Cycloporus papillosus*. **A**–**D** dorsal views of living animals **E** sagittal reconstruction of a whole specimen **F** sagittal reconstruction of the copulatory apparatus. Anterior to the right in **E**, **F.**

Elongated worms 11 mm long and 6 mm wide. Body shape elongated with light undulating margins and rounded anterior and posterior ends. Dorsal surface with numerous papillae. Colouration orange, yellowish orange or translucent grey with white patches at the mid-dorsal line (Figure 1A, B, C, D). Ventral side smooth and pale. Short inconspicuous marginal tentacles. Sucker located approximately in the middle of the body (Figure 1E). Tentacular eyes scattered over dorsal margin of tentacles (Figure 1A), cerebral eyes in two elongated, anteriorly anastomosing clusters. Plicate cylindrical or tubular pharynx near anterior end, frontally oriented; oral pore posterior to brain. Male and female genital pores clearly separated and posterior to pharynx (Figure 1E).

Male copulatory apparatus located posterior to male pore and oriented mainly dorso -ventrally, but also directed frontally (Figure 1E, F). Male system consists of a short, armed (stylet) penis papilla, a true prostatic vesicle with a smooth glandular epithelium, and a well developed, muscularized seminal vesicle. Prostatic vesicle opens directly into penis papilla, and seminal vesicle empties directly into distal end of prostatic vesicle. Vasa deferentia, sometimes very dilated, open proximally through a common duct (common vas deferens) into seminal vesicle.

Inconspicuous female system lies posterior to the male pore and is characterized by a short female atrium, female duct (vagina), and characteristic uterine vesicles. Abundant cement glands are located around female pore and distal part of vagina.

####### Remarks on biology.

*Cycloporus papillosus* is a natural predator of *Botrylloides violaceus* Oka, 1927 (Ascidiacea), which is a clear example of an invasive species. *Botrylloides violaceus* grows on all types of substrates, including other living animals such as mussels, small sea cucumbers or other ascidians, covering them completely and killing them. *Botrylloides violaceus* has completely replaced *Botrylloides leachii*, the autochthonous ascidian in this area. Both species of ascidians compete for the same substrate. *Cycloporus papillosus* preys on *Botrylloides violaceus* and places its egg plates (Fig. 1D) in the folds of this species (in the area where new zooids grow and extend the colony) or under the unattached colony, thereby ensuring larval protection and the availability of food after hatching.

####### Distribution.

In Galicia, three specimens of *Cycloporus papillosus* were captured from mussels collected on *Botrylloides violaceus* on the docks of the Yacht Club Ribeira (Ria de Arosa, Galicia, Spain). Depth varied between 0.5 and 1 metres (42°33.7770N, 008°59.2970W; 42°33.7850N, 008°59.3140W; 42°33.7930N, 008°59.3290W). Another specimen (Figure 1B) was collected on a colony of *Botryllus schlosseri* (Ascidiacea) growing on a rock of the island of Rua (Ria de Arosa, Galicia, Spain), at a depth of 14 metres (42°32.9650N, 008°56.4590W).

This is the southernmost European record for *Cycloporus papillosus*, and the first for the Atlantic coast of the Iberian Peninsula. Other localities from where this polyclad has been reported are: Bergen, Norway (Jensen 1878); Rovigno, Croatia (Vàtova 1928); Susaki near Simoda, Japan (Kato 1937); Porto Praia, Cape Verde (Laidlaw 1906); Plymouth, United Kingdom (Gamble 1893).

##### Genus *Eurylepta* Ehrenberg, 1831

###### Eurylepta cornuta

(O.F. Müller, 1776) Ehrenberg, 1831

http://species-id.net/wiki/Eurylepta_cornuta

Figure 2

####### Material examined.

Two mature specimens captured during winter 2012 (15/01/2012). Voucher: one specimen sectioned sagittally, stained with Azan and deposited in the Invertebrate Collections of the MNCN; Cat. Nr: MNCN 4.01/626 to 4.01/647 (22 slides).

####### Description.

**Figure 2.**
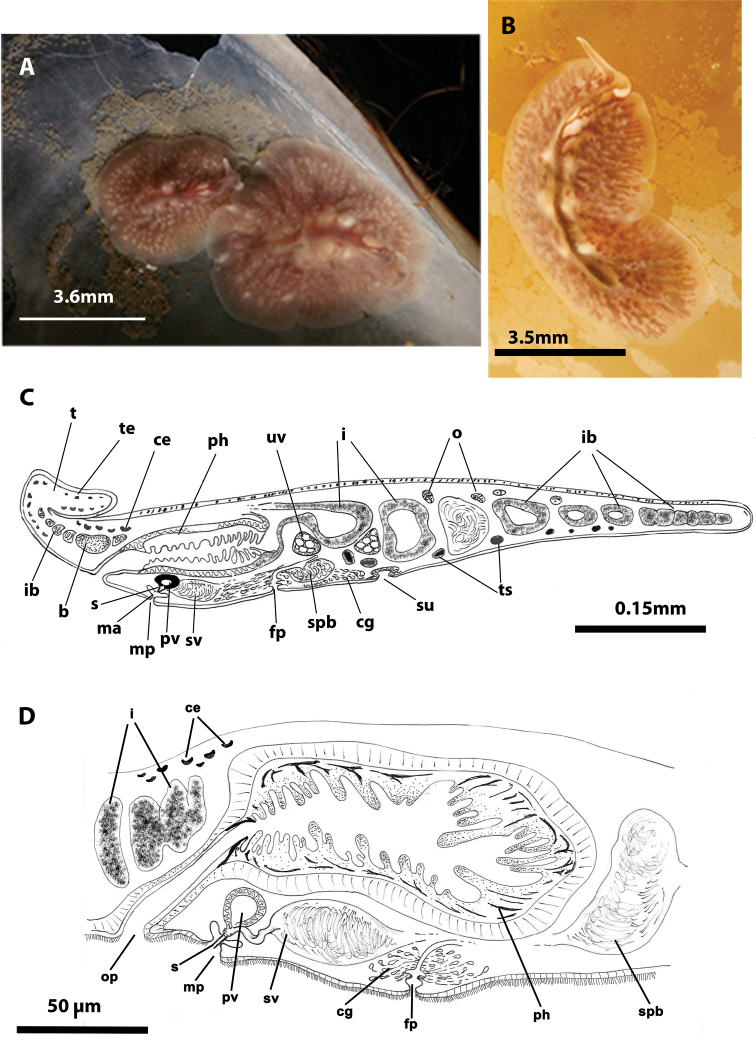
*Eurylepta cornuta*. **A**, **B** dorsal views of living animals **C** sagittal reconstruction of a whole specimen **D** sagittal reconstruction of the copulatory apparatus. Anterior to the left in **C**, **D.**

Captured worms 10 mm long and 5 mm wide. Body shape elongated, with straight margins. Dorsal surface smooth. Background coloration of the dorsal surface pale brown, translucent, with dark branched bands, red or brown, depending on intestinal contents, (Figure 2A). Ventral side pale yellow without bands. With narrow conical marginal tentacles; sucker slightly posterior to the middle of the bodies. Tentacular eyes at the base of the tentacles (Figure 2C) and cerebral eyes in two elongated clusters, sometimes extending over the pharynx. Tubular, whitish pharynx is visible at the anterior end (Figure 2C).

Male copulatory apparatus located posterior to male pore and directed forwards (Figure 2D). Prostatic vesicle oriented antero-dorsally, with a smooth glandular epithelium, and directly connected to tubular stylet of penis papilla. Seminal vesicle empties through a narrow short duct into distal end of prostatic vesicle. Female pore lies closely behind male pore, but is clearly separated. Female atrium elongated. Cement pouch rounded and followed by a short vagina and opening of uteri. A pair of uterine vesicles is present.

####### Remarks.

*Eurylepta cornuta* shows two varieties: 1. *Eurylepta cornuta* var. *lobianchi*, first described by Lang (1884) and known from the Mediterranean Sea, and 2. *Eurylepta cornuta* var. *melobesiarum*, first described by Schmidtlein (1880) as *Proceros melobesiarum*. The main difference between these varieties is in the arrangement of the cerebral eyes (Lang 1884; Bock 1913). In *Eurylepta cornuta* var. *melobesiarum* the elongated patches are shorter than in *Eurylepta cornuta* var. *lobianchi*. Therefore, and in agreement with other authors (e.g., Micoletzky 1910; Bock 1913; Faubel 1984), we consider the difference not enough to maintain the two varieties and propose that should no longer be recognized.

####### Distribution.

*Eurylepta cornuta* was found in empty shells of *Mytilus galloprovincialis* (Figure 2A), which were attached to mussel culture ropes suspended from specially designed rafts called “bateas”, located in La Palmeira (Ria de Arosa, Galicia, Spain) at a depth of 13 metres (42°34.3910N, 008°56.6360W). Several specimens of *Eurylepta cornuta* (Figure 2B) were also captured for the first time within *Saccorhiza polyschides* stipes (macroalgae), at a depth of 8 metres in “Cuberto Camouco” (Ria de Arosa, Galicia, Spain) (42°33.4150N, 008°57.8390W). Another specimen was found under a stone on the island of Rua, at a depth of 14 metres (42°32.9200N, 008°56.4220W).

*Eurylepta cornuta* has been known since the 18^th^ century from Kristiansand, Norway (O.F. Müller 1776) and since the 19^th^ century from Belfast Bay, Ireland (Thompson 1845); Saint Malo, France (Keferstein 1868); Plymouth Sound, United Kingdom (Gamble 1893); and the Gulf of Naples, Italy (Lang 1884).

##### Genus *Euryleptodes* Heath & McGregor, 1912

###### Euryleptodes galikias sp. n.

http://zoobank.org/7D732693-EB62-4A44-9958-FC589550BFEE

http://species-id.net/wiki/Euryleptodes_galikias

Figure 3

####### Material examined.

One specimen captured during winter 2012 (09/12/2012).

**Holotype.** One sagittally sectioned specimen, stained with Azan and deposited in the Invertebrate Collections of the MNCN; Cat. Nr. MNCN 4.01/502 to 4.01/572 (71 slides).

####### Type locality.

Ribeira (Ria de Arosa, Galicia, Spain). Depth; 5 metres (42°33.7760N, 008°59.3390W).

####### Description.

**Figure 3.**
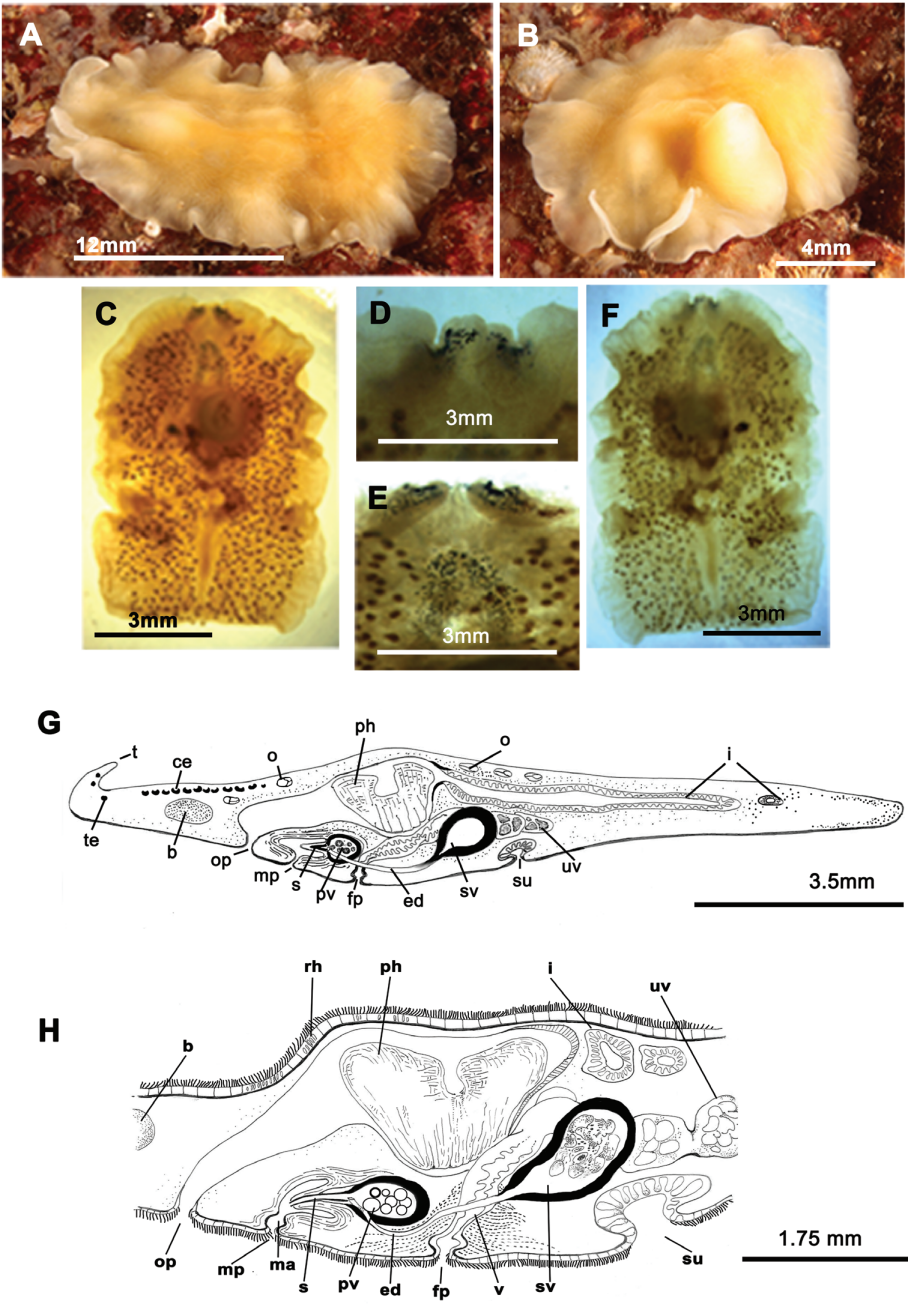
*Euryleptodes galikias*. **A**, **B** dorsal view of a living animal **C** dorsal and **F**, ventral views of a fixed specimen **E** dorsal and **D** ventral details of the eyes **G** sagittal reconstruction of a whole specimen **H** sagittal reconstruction of the reproductive system **H.** Anterior to the left in **A**, **F** and **G.**

Living elongated worms 25 mm long and 14.4 mm wide. Body shape broad, slightly oval and with undulating margins. Colouration in living animals yolk yellow (Figure 3A, B); fixed individuals have a transparent look; with small dark patches representing the uterine network over entire body surface (Figure 3C). Ventral side pale yellow. Marginal tentacles well-developed, conical. Sucker posterior to middle of the body. Tentacular eyes on the dorsal margin of tentacles (Figure 3D), marginal eyes in two small clusters on anterior margin (Figure 3E) and cerebral eyes in two elongated anteriorly anastomosing clusters (Figure 3E). Tubular pharynx near anterior end; oral pore closely posterior to brain. Male and female genital pores clearly separated, anterior and ventral to the pharynx, respectively (Figure 3F).

Male copulatory apparatus located posterior to the male pore and directed forwards (Figure 3G, H). Male system consists of a small penis papilla with a short stylet, a true prostatic vesicle with a smooth glandular epithelium and an elongated seminal vesicle. Prostatic vesicle is oval and small, and opens directly into the penis papilla, which projects into the male atrium. Seminal vesicle empties into distal end of prostatic vesicle through a long, narrow ejaculatory duct. Characteristic spermiducal vesicles open proximally into seminal vesicle.

Small female system is difficult to distinguish except for uterine network and well developed cement glands. Female pore lies behind male pore and is clearly separated from it. Vagina shows a small expansion (or cement pouch; cf Hyman 1953), ascends dorsally and subsequently splits into two uteri. Uteri extend as a uterine network with channels that connects to the ovaries. Uterine vesicles absent.

####### Discussion.

Based on the presence of the conical marginal tentacles, the spermiducal vesicles, an armed penis and a uterine network, this new species belongs to the genus *Euryleptodes* Heath & McGregor, 1912 of the family Euryleptidae Lang, 1884. The genus *Euryleptodes* presently comprises of two species: *Euryleptodes cavicola* Heath & McGregor, 1912 and *Euryleptodes insularis* Hyman, 1953, both from California.

*Euryleptodes galikias* sp. n. differs from *Euryleptodes insularis* in the long ejaculatory duct, the tentacular eyes over the tentacles, and the frontal marginal eyes.

Compared to *Euryleptodes cavicola*, which has a long stylet, the stylet of *Euryleptodes galikias* is short. Furthermore, *Euryleptodes cavicola* lacks spermiducal vesicles, thus distinguishing it from both *Euryleptodes galikias* and *Euryleptodes insularis*.

Differences in colouration are also apparent between the species: yolk yellow in *Euryleptodes galikias* sp. n., greenish white in *Euryleptodes cavicola*, and brown with dark spots in *Euryleptodes insularis*. Lastly, the pattern of distribution is vastly different, with *Euryleptodes galikias* off the eastern coast of the North Atlantic Ocean (Spain), while the other two species occur off the eastern coast of the North Pacific Ocean (California).

####### Distribution.

*Euryleptodes galikias* sp. n. was found within empty shells of *Mytilus galloprovincialis*, which were attached to a boat anchor on the third dock of the Yacht Club Ribeira (Ria de Arosa, Galicia, Spain), of a depth of 5 metres (42°33.7760N, 008°59.3390W).

##### Genus *Prostheceraeus* Schmarda, 1859

###### Prostheceraeus vittatus

(Montagu, 1815) Lang, 1884

http://species-id.net/wiki/Prostheceraeus_vittatus

Figure 4

####### Material examined.

Two specimens captured in winter 2010 and 2012 (15/03/2010 and 24/02/2012). Voucher: one specimen sectioned sagittally, stained with Azan and deposited in the Invertebrate Collections of the MNCN; Cat. Nr: MNCN 4.01/648 to 4.01/662 (15 slides).

####### Description.

**Figure 4.**
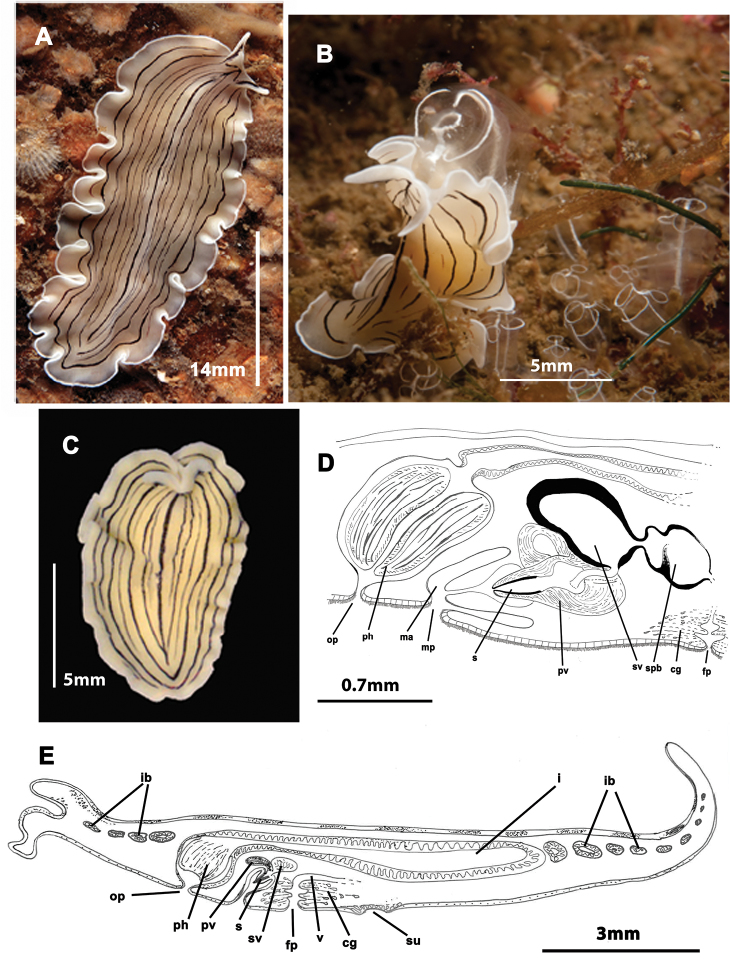
*Prostheceraeus vittatus*. **A** dorsal view of a living animal **B** living animal feeding on ascidians **C** dorsal view of a fixed specimen **D** sagittal reconstruction of the copulatory apparatus **E** sagittal reconstruction of a whole specimen. Anterior to the left in **D**, **E.**

Elongated worms 10–30 mm long and 7–15 mm wide. Body shape elongated, leaf-shaped, with pointed anterior and posterior ends, and with light undulating margins. Marginal tentacles well developed with whitish edges and pointed ends. Dorsal surface smooth. Background coloration whitish or ivory, with black, continuous stripes; between the stripes, black discontinuous lines are present (Figure 4C). Faint white band runs along the entire body margin (Figure 4A, B). Ventral side smooth and pale. Sucker in middle of body or slightly more posterior (Figure 4E). Cerebral eyes form two compact elongated, frontally anastomosing groups (Figure 4A). Tentacular eyes scarce and mainly at base of tentacles. Tubular pharynx near anterior end, oral pore in posterior region first quarter of the body. Male and female genital pores clearly separated and located behind the pharynx (Figure 4D, E).

Male copulatory apparatus with antero-dorsally oriented prostatic vesicle (Figure 4D, E). Male system consists of a short penis papilla armed with a small conical stylet, a true prostatic vesicle with a smooth glandular epithelium and a seminal vesicle with a thick muscle layer. Vasa deferentia join a dilated common vas deferens that opens into seminal vesicle. Copulatory complex lies forwardly oriented, and seminal vesicle opens through a small duct directly into distal end of prostatic vesicle.

Female system lies posterior to male pore and is characterized by a short, rounded female atrium and a cement duct or pouch. In our specimen, a second dilatation (so-called shell gland pouch) follows the atrium, into which shell glands open. Posteriorly-orientated vagina and numerous uterine vesicles are situated medially to this pouch.

####### Biology.

*Prostheceraeus vittatus* feeds mainly on *Clavelina* ascidians, as other *Prostheceraeus* species (Figure 4B).

####### Remarks.

Lang (1884) mentions in the original description that specimens less than 1.5–2 cm in length were immature. However, sometimes our specimens from the Atlantic coast were mature, despite their small size (c. 1.5 cm in length).

####### Distribution.

Two specimens of *Prostheceraeus vittatus* were captured during this study. The first animal was collected from “A Tiñosa” (Ria de Arosa, Galicia, Spain) on a rocky bottom between *Clavelina lepadiformis* colonies, at a depth of 24 metres (42°32.8240N, 008°57.9920W). The other worm was found on stones in “Petón Bajo” (Ria de Arosa, Galicia, Spain), at a depth of 16 metres (42°32.9880N, 008°57.9920W).

*Prostheceraeus vittatus* is known from the North Atlantic coasts of the United Kingdom, France, Ireland, Scandinavia, Norway, Denmark, from the Mediterranean shores in Italy (Gulf of Naples) (Faubel and Warwick 2005) and Spain (Catalonia) (Novell 2003). This is the first record for the species from the North Atlantic side of the Iberian Peninsula.

###### Prostheceraeus moseleyi

Lang, 1884

http://species-id.net/wiki/Prostheceraeus_moseleyi

Figure 5

####### Material examined.

Two specimens captured during the spring of 2010 (07/06/2010). Vouchers: one specimen sectioned sagittally, stained with Azan and deposited in the Invertebrate Collections of the MNCN: Cat. Nr: MNCN 4.01/663 to 4.01/688 (26 slides); one specimen sectioned sagittally, stained with Azan and deposited in the Invertebrate Collections of the MNCN: Cat. Nr: MNCN 4.01/689 to 4.01/731 (43 slides).

####### Description.

**Figure 5.**
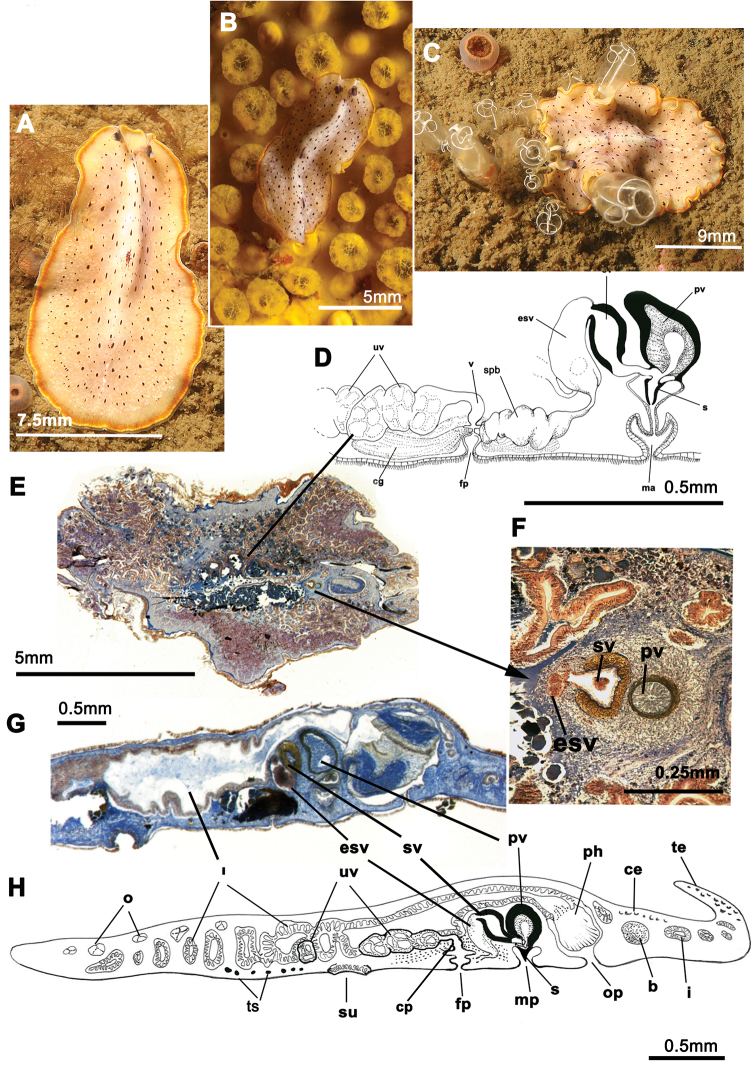
*Prostheceraeus moseleyi*. **A**, **B**, **C** dorsal views of living animals **D** sagittal reconstruction of the reproductive system **E** dorso-ventral histological sections of the whole animal **F** dorso-ventral histological sections of the copulatory apparatus **G** sagittal histological section in the region of the pharynx and copulatory apparatus **H** sagittal reconstruction of a whole specimen. Anterior to the right in **C**, **D**, **E**, **F**, **G** and **H.**

Elongated worms 1–1.5 cm long and 0.5–0.7 mm wide (Figure 5A, B, C). Body shape elongated, wider at the posterior end, with light undulating margins and rounded anterior and posterior ends. Marginal tentacles with characteristic purple pigment. Dorsal surface smooth. Dorsal colouration whitish or yellowish, with dark, rounded spots and a yellow band along the body margin; at times background pigmentation faintly orange or pinkish (Figure 5A, B). Ventral side smooth and pale. Sucker approximately in middle of body. Tentacular eyes scarce and scattered over tentacles; cerebral eyes inconspicuous, in two elongated rows. Plicate cylindrical or tubular pharynx near anterior end, oriented frontally; oral pore behind brain. Male and female genital pores clearly separated and behind pharynx (Figure 5H).

Male copulatory apparatus perpendicular to male pore (Figure 5D, G, H). Male system consists of a short penis papilla, armed with a small conical stylet, a true prostatic vesicle with a smooth glandular epithelium, and a seminal vesicle with a thick muscle layer. Vasa deferentia, sometimes very dilated, join an expanded vas deferens (possibly an external seminal vesicle; Figure 5F, G) before opening directly into true seminal vesicle. Prostatic vesicle opens at tip of penis papilla and seminal vesicle opens through a duct into distal end of prostatic vesicle.

Female system (Figure 5D, E) lies posterior to male pore and is characterized by a short, rounded female atrium, a cement duct or pouch, followed by a second dilatation of atrium and a posteriorly-orientated vagina. Cement and shell glands empty into cement duct.

####### Biology.

*Prostheceraeus moseleyi* feeds mainly on *Clavelina lepadiformis* (Ascidiacea) (Figure 5B, C).

####### Remarks.

Faubel (1984) considers *Prostheceraeus moseleyi* as a species *incertae sedis* because in the original description by Lang (1884) the uterine vesicles were not mentioned. In our specimens, multiple uterine vesicles (Figure 5D, E) run along both sides of the main body axis and therefore, *Prostheceraeus moseleyi* can now be considered as a valid species of the genus *Prostheceraeus*.

####### Distribution.

Specimens of *Prostheceraeus moseleyi* were collected from the bottom of the pier at the Yacht Club Ribeira (Ria de Arosa, Galicia, Spain) at a depth of 5 to 10 metres (42°33.7410N, 008°59.3380W). This species was recorded from Italy (Gulf of Naples, Lang 1884) and Spain (in several localities of the coast of Catalonia, Novell 2003). Our sample represents the first record for the Atlantic coast and outside from the Mediterranean Sea.

#### Family PROSTHIOSTOMIDAE Lang, 1884

##### Genus *Prosthiostomum* Quatrefages, 1845

###### Prosthiostomum siphunculus

(Delle Chiaje, 1822) Lang, 1884

http://species-id.net/wiki/Prosthiostomum_siphunculus

Figure 6

####### Material examined.

Two specimens collected in winter 2012 (09/01/2012). Voucher: one specimen sectioned sagittally, stained with Azan and deposited in the Invertebrate Collections of the MNCN Cat. Nr: MNCN 4.01/732 to 4.01/744 (13 slides).

####### Description.

**Figure 6.**
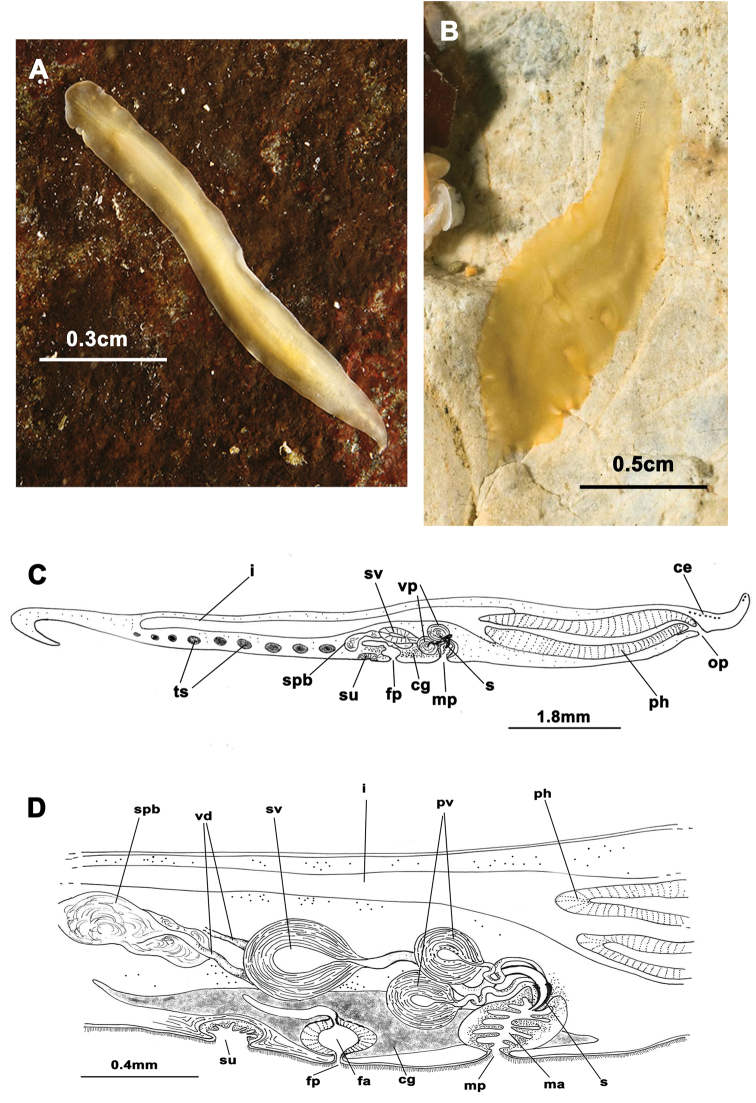
*Prosthiostomum siphunculus*. **A**, **B** dorsal views of living animals **C** sagittal reconstruction of a whole specimen **D** sagittal reconstruction of the copulatory apparatus. Anterior to the right in figures **C**, **D.**

Mature, elongated specimens 10–18 mm long, occasionally as long as 30 mm, and 4–6 mm wide. Body shape long and narrow, sometimes with a little fan-like expansion at the anterior end, straight margins, rounded anterior and tapered posterior end. Without tentacles. Dorsal surface smooth. Dorsal colouration beige to yellow, without spots or bands (Figure 6A, B). Ventral side smooth and pale. Sucker in the middle of the body or slightly anterior. Cerebral eyes arranged in two slightly curved rows; marginal eyes along the anterior edge; young individuals have an eye-free medial area. Well-developed plicate tubular pharynx located near the anterior end, oriented frontally; oral pore closely behind the brain. Male and female genital pores separated and located posterior to pharynx (Figure 6C).

Male system consists of a short penis papilla armed with a conical, Arabian dagger-like stylet an anterior orientated seminal vesicle and two spherical prostatic vesicles with a smooth glandular epithelium covered with a thick muscle layer. Vasa deferentia open separately into a seminal vesicle. Ejaculatory duct and prostatic ducts very long and spiral-shaped; prostatic ducts meet at proximal end of small penis papilla. Male atrium elongated and conical (Figure 6D).

The female system lies posterior to male pore and is characterized by a short, spindle-like female atrium, a cement gland pouch, followed by a backwards orientated vagina, and backwards directed uteri.

####### Distribution.

One specimen was found in mussel samples collected from the floats of the third dock of the Yacht Club Ribeira at a depth of 1 metres (42°33.7700N, 008°59.3260W). The other animal was collected from under a rock in the area “A Ameixida” at a depth of 6 metres (42°32.2490N, 008°59.1640W). *Prosthiostomum siphunculus* is known from the western European Atlantic coasts, the Mediterranean and the Tyrrhenian Sea, and also from North and South Africa, Somalia and Vietnam (Prudhoe 1985).

### Other species of Polycladida found in the study area

The following four well-known species were identified on the basis of their characteristic external anatomy and were also photographed:

#### Family EURYLEPTIDAE Lang, 1884

##### Prostheceraeus roseus

Lang, 1884

http://species-id.net/wiki/Prostheceraeus_roseus

Figure 7A, B

###### Remarks.

*Prostheceraeus roseus* belongs to the family Euryleptidae and is characterized by the following: an elongated, oval body shape and smooth surface; conspicuous marginal tentacles; cerebral eye clusters in two small, parallel rows that do not anastomose. This species is readily recognizable by its distinct pink to purple pigmentation with white longitudinal stripes and a white edge that runs along the entire body margin. The tubular, bell-shaped pharynx oriented frontally, and the digestive system well developed with numerous, anastomosing branches. The reproductive system shows the characteristics of the genus: male copulatory apparatus frontally oriented; penis armed with tubular pointed stylet and female complex with multiple uterine vesicles.

**Figure 7.**
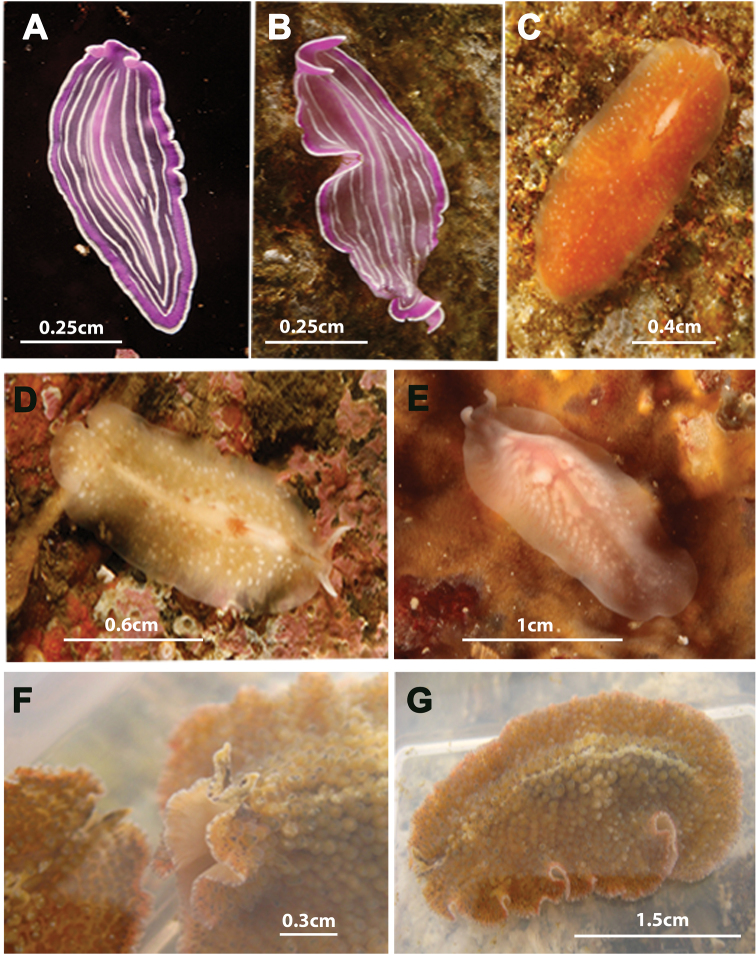
Species photographed but not collected. **A**, **B** *Prostheceraeus roseus* **C** *Stylostomum ellipse* **D**, **E** *Oligocladus sanguinolentus* **F**, **G** *Thysanozoon brocchii*.

###### Distribution.

*Prostheceraeus roseus* was one of the first species collected during this study, first in summer of 2010, then in autumn of 2013 (14/05/2010 and 10/10/2013). Two specimens were captured in the mouth of the Ria de Arosa in Corrubedo: one on a rocky wall in “Canteiro”, at a depth of 27 metres (42°30.5540N, 009°05.1260W), and the other on *Pycnoclavella producta* (Ascidiacea) in “A Dianteira”, at a depth of 23 metres (42°30.9100N, 009°04.3750W). *Prostheceraeus roseus* is also known from the Gulf of Naples, Italy (Lang 1884). These findings represent the first record for the Atlantic coast outside the Mediterranean Sea.

##### Stylostomum ellipse

(Dalyell, 1853) Lang, 1884

http://species-id.net/wiki/Stylostomum_ellipse

Figure 7C

###### Remarks.

*Stylostomum ellipse* belongs to the family Euryleptidae and is characterized by: a small to moderate size and oval outline, sometimes pear-shaped; dorsal surface smooth; marginal tentacles very reduced; small cerebral and marginal eye clusters. *Stylostomum ellipse* is also characterized by the common opening of the male and oral pore, located in front of the pharynx. In contrast, the female pore is situated behind the pharynx. The penis papilla is armed with a tubular pointed stylet and the female system has two uterine vesicles.

###### Distribution.

Only one specimen of *Stylostomum ellipse* was captured (09/01/2012) from stones in the “O Rodal de Nuestra Señora”, at a depth of 32 metres (42°31.9010N, 008°58.5330W). *Stylostomum ellipse* is considered a cosmopolitan species. Records of *Stylostomum ellipse* are distributed around the world including the Mediterranean Sea, Atlantic coast of western Europe (Great Britain, France, Scandinavia), South America (Falkland Island, Tierra de Fuego), South Africa (Cape Town) (Prudhoe 1985) and Antarctica (Hallez 1905). Our samples represent the first record for the Iberian Peninsula.

##### Oligocladus sanguinolentus

(Quatrefage, 1845) Lang, 1884

http://species-id.net/wiki/Oligocladus_sanguinolentus

Figure 7D, E

###### Remarks.

*Oligocladus sanguinolentus* belongs to the family Euryleptidae and is characterized by an elongate body with a translucent appearance, thus making the intestinal contents visible and giving the animal a bloodied appearance. The marginal tentacles are long, narrow and clearly separated. The tentacular eyes scattered at the base of the tentacles; and the cerebral eyes in two elongated, diffuse clusters. The oral pore lies in front of the brain. An anal pore (Lang 1884) that opens at the dorsal surface was not observed. Male complex with a penis papilla armed with a tubular, pointed stylet. Female apparatus with multiple uterine vesicles.

###### Distribution.

Two specimens were found: one on stones in the outer breakwater of the Yacht Club Ribeira at a depth of 8 metres (42°33.7760N, 008°59.2740W), and the other in “Torre de Abajo” at a depth of 28 metres (42°32.7150N, 008°57.0950W).

*Oligocladus sanguinolentus* is known from the coastal and littoral shores of Saint Malo (France), Isle of Man, Scilly Islands, various sites of Great Britain (United Kingdom), Gibostad (Norway) and Porto Grande de Sao Vicente (Cape Verde archipelago) (Faubel and Warwick 2005). The only record for the Mediterranean Sea is in the Gulf of Naples (Italy) (Lang 1884).

#### Family PSEUDOCEROTIDAE Lang, 1884

##### Thysanozoon brocchii

(Risso, 1818) Grube, 1840

http://species-id.net/wiki/Thysanozoon_brocchii

Figure 7F, G

###### Remarks.

*Thysanozoon brocchii* belongs to the family Pseudocerotidae and is characterized by an oval, oblong body shape; marginal tentacles with eyes; a single pair of eye clusters which anastomose frontally (Figure 7F). The dorsal surface covered with characteristic acorn-like papillae, which may house small intestinal branches (Figure 7G). Reproductive system with the characteristics of the genus: i.e. a paired male apparatus with a seminal vesicle and armed penis papillae, a prostatic vesicle oriented antero- or medio-dorsally to the ejaculatory duct and a female apparatus with branched uteri.

###### Distribution.

One specimen of *Thysanozoon brocchii* was found under a rock in the estuary of Muros in Punta Insuela (Galicia, Spain) (10/05/2010) at a depth of 5 metres (42°46.8550N, 009°00.2610W). *Thysanozoon brocchii* is considered to be cosmopolitan with records from the Atlantic Ocean in South America, Peninsula Valdes (Brusa et al. 2009), Mar del Plata (Bulnes et al. 2011), Ilha de Sao Sebastiao, Ilha das Palmas; North America (Florida), Cape Verde Islands and South Africa; it is also recorded from the Pacific Ocean in Japan and Hawaii, and from the Mediterranean and Adriatic seas, including the Suez Canal (Prudhoe 1985). For the Atlantic coast of the Iberian Peninsula, our collections represent the first record.

## General discussion

Previous polyclad studies of the North Atlantic Ocean coasts (Bock 1913, Prudhoe 1985, Faubel and Warwick 2005) suggested that the Iberian Peninsula may have a great diversity of species belonging to this group of flatworms, however the actual level of diversity in this region was unknown. This study describes, for the first time, species of the Order Polycladida from the Ibero-Atlantic environment. These records reconfirm the broad geographic range for some polyclad species like *Thysanozoon brocchii*, *Stylostomum ellipse*, *Prosthiostomum siphunculus* or *Oligocladus sanguinolentus*. The species collected can be divided into two groups according to their distribution.

Group 1: includes species with a cosmopolitan distribution that are known from the Atlantic Ocean and the Mediterranean Sea: *Prosthiostomum siphunculus*, *Thysanozoon brocchii* and *Stylostomum ellipse*.

Group 2: includes species with a distribution restricted to the European, North Atlantic and/or Mediterranean coasts and includes *Eurylepta cornuta*, *Oligocladus sanguinolentus*, *Prostheceraeus vittatus*, *Prostheceraeus roseus* and *Prostheceraeus moseleyi*. The two last-mentioned species had previously been known only from the Mediterranean Sea and are here reported for the first time from the Atlantic Ocean.

*Euryleptodes galikias* sp. n., is new to science, and its distribution is currently limited to the Galician Atlantic coast of the Iberian Peninsula.

In summary and based on the results, the distribution range of described polyclads (hitherto limited to the North Atlantic or the Mediterranean basin) has been expanded to the Atlantic shores of the Iberian Peninsula (Lusitanean Region).

## Supplementary Material

XML Treatment for Cycloporus papillosus

XML Treatment for Eurylepta cornuta

XML Treatment for Euryleptodes galikias

XML Treatment for Prostheceraeus vittatus

XML Treatment for Prostheceraeus moseleyi

XML Treatment for Prosthiostomum siphunculus

XML Treatment for Prostheceraeus roseus

XML Treatment for Stylostomum ellipse

XML Treatment for Oligocladus sanguinolentus

XML Treatment for Thysanozoon brocchii
